# Symptomatic Vaginal Infection by *Neisseria meningitidis* Resulting in Meningitis with Septic Shock

**DOI:** 10.5811/cpcem.2019.3.41750

**Published:** 2019-03-27

**Authors:** Ryan Offman, Elissa Boggs, Adam Gwizdala

**Affiliations:** * Mercy Health-Muskegon, Department of Emergency Medicine, Muskegon, Michigan; † Michigan State University College of Osteopathic Medicine, Department of Osteopathic Medical Specialties, East Lansing, Michigan; ‡ Mercy Health-Muskegon, Department of Pharmacy, Muskegon, Michigan

## Abstract

The most common infectious etiologies of vaginitis include Gardnerella bacterial vaginosis, candidiasis, and trichomoniasis. A few case reports describe symptomatic infection with *Neisseria (N) meningitidis*, an organism with potential for causing systemic disease with a high rate of morbidity and mortality. We describe a patient who presented with fulminant meningitis secondary to symptomatic vaginitis in which *N. meningitidis* was cultured. Due to the potential for significant morbidity and mortality as demonstrated by this case report, knowledge of this entity may prompt physicians to aggressively treat patients with vaginal cultures that are positive for *N. meningitidis*.

## INTRODUCTION

Vaginitis is a common diagnosis among women, and in 40–50% of cases, *Gardnerella vaginalis* is isolated. This is followed by candidiasis, which causes 20–25% of cases, Trichomoniasis causes 15–20% of cases, and non-infectious causes account for 5–10% of cases.^1^ *Neisseria (N) meningitidis* has been isolated from the urogenital region of women with symptomatic vaginitis previously, although no current treatment recommendations exist.^2^ Although a very rare isolate from the urogenital region, *N. meningitidis* has been documented to cause both self-limiting vaginitis, arthritis, and meningococcemia.^3^,^4^ However, a literature search revealed no case reports of symptomatic vaginitis secondary to *N. meningitidis* leading to fulminant meningitis with septic shock.

Meningococcemia is a rapidly progressive infection mostly seen in young, healthy individuals who live in crowded environments.^5^ In the United States (U.S.), there are about 1,000–3,000 new cases diagnosed each year, with a prevalence between 0.5 and 1.1 cases per 100,000 people, with an associated mortality rate of up to 53%.^6^ We present a case in which *N. meningitidis* was isolated from vaginal cultures during work-up for symptomatic vaginitis followed by classic *N. meningitidis* meningitis presenting with septic shock. Recognition and treatment of *N. meningitidis* from uncommon sources may require more aggressive treatment than previously thought.

## CASE REPORT

A 72-year-old female with a past medical history of hypertension presented to the emergency department (ED) with one day of generalized weakness, headache and rash. Review of her medical history revealed that she presented to her primary care physician with complaints of vaginal discharge two weeks prior to her presentation to the ED. Despite treatment with metronidazole, symptoms persisted prompting a speculum exam and vaginal cultures obtained by primary care physician five days prior to her ED encounter. These returned positive for *N. meningitidis*. At the time of presentation, she had not followed up for those results and therefore had no further treatment. The patient had been in her normal state of health until the previous evening when she described general malaise and fell asleep early. Her husband stated that she also slept much later than usual and when he tried to wake her, she seemed lethargic. Review of systems was otherwise negative.

Initial vital signs revealed a temperature of 101.7 degrees Fahrenheit, a pulse of 120 beats per minute, respiratory rate of 24 breaths per minute, and a blood pressure of 107/49 millimeters of mercury. The patient was found to be ill-appearing and obtunded. She had nuchal rigidity and a diffuse, non-blanching, purpuric rash. Other than atrial fibrillation with a rapid ventricular response, the remaining physical exam was unremarkable.

Immediate concern for meningococcemia prompted empiric vancomycin (20 milligrams per kilogram, intravenous [mg/kg, IV]), ceftriaxone (2 grams [g], IV), ampicillin (2 g IV), dexamethasone (10 mg IV), and an initial 2-liter (L) normal saline bolus. After an unremarkable non-contrast computed tomography of her head, a lumbar puncture was performed revealing turbid cerebral spinal fluid with a white blood cell count of 262 cells per millimeter cubed (mm^3^) (0–5 cells/mm^3^), glucose of 28 mg per deciliter (dL) (40–70 mg/dL), and a protein of 155 mg/dL (15–45mg/dL). Gram stain showed many gram-negative diplococci. Her initial serum white blood cell count was 7.4 thousand cells/mL^3^ (4.0–10.0 cells/mL^3^) and the initial lactic acid was 8.1 millimoles per liter (mmol/L) (0.5–1.6 mmol/L). Despite aggressive fluid resuscitation, the patient rapidly decompensated requiring central line placement, intubation, and norepinephrine infusion.

The patient was admitted to the intensive care unit where she had persistent septic shock that required corticosteroid administration and cardiovascular support with norepinephrine, epinephrine, and vasopressin. Her hospital stay was further complicated by disseminated intravascular coagulopathy and ischemic hepatitis. Cultures from blood and cerebral spinal fluid grew *N. meningitidis*, which was sensitive to ceftriaxone. She received a seven-day course upon recommendation of infectious disease specialists. On hospital day 10, she was discharged to a long-term acute care facility with ischemic necrosis of the digits of her hands and feet bilaterally, which would later require operative intervention.

## DISCUSSION

We present a rare case in which a patient presented with a seemingly innocuous presentation of vaginitis two weeks prior to development of meningitis with septic shock secondary to *N. meningitidis*. Vaginitis is often encountered in the ED, and emergency physicians must know appropriate treatment plans as well as proper follow-up on culture results obtained on prior emergency visits, or from outpatient physicians.

The most common infectious etiologies of vaginitis include Gardnerella bacterial vaginosis, candidiasis, and trichomoniasis.^1^ Bacterial vaginosis is a polymicrobial infection with a predominance of *Gardnerella vaginalis* occurring when dominance of the normal flora is disrupted.^7^ While not a sexually transmitted disease, sexual activity and other lifestyle choices do play a role as risk factors include multiple sexual partners, lack of condom usage, cigarette smoking, and douching.^8^,^9^ Candidiasis is a fungal infection that occurs with disturbances in vaginal flora, vaginal pH or glycogen stores of the vaginal epithelial cells. Risk factors include treatment with systemic antibiotics, presence of menstrual blood or semen, hormone therapy (including oral contraceptives), pregnancy, immunosuppression, and diabetes mellitus.^10^ Sexual transmission is also possible.^11^

**CPC-EM Capsule**

What do we already know about this clinical entity?

*It is known that Neisseria (N) meningitidis can cause localized genitourinary infections and occasionally mild systemic illness*.

What makes this presentation of disease reportable?

*Although there have been cases of mild systemic illness secondary to N. meningitidis reported, infection leading to septic shock has never been described*.

What is the major learning point?

*Treatment of genitourinary infections secondary to N. meningitidis requires aggressive therapy and prompt follow up*.

How might this improve emergency medicine practice?

*Knowledge of this entity could prevent significant morbidity and mortality as emergency physicians are often tasked with following up on cultures*.

Trichomoniasis is a sexually transmitted disease caused by the protozoan *Trichomonas vaginalis*. Incidence increases with age. It is more prevalent in lower socioeconomic classes, those with increased number of lifetime sexual partners, and earlier age of onset of sexual activity.^12^ Up to 75–80% of infections can be asymptomatic, aiding its transmission.^13^ Treatment is imperative as trichomoniasis is associated with multiple pregnancy complications, pelvic inflammatory disease, and increased transmission of other sexually transmitted diseases such as human immunodeficiency virus (HIV).

Treatment of infectious vaginitis varies by clinical scenario. Bacterial vaginitis can be treated with oral metronidazole, clindamycin, or tinidazole. Topical regimens include metronidazole or clindamycin. It is recommended that all symptomatic pregnant patients be treated with oral metronidazole or clindamycin. Candida vaginitis can be treated by all of the azole class of antifungals, including single-dose fluconazole. Treatment for trichomoniasis consists of oral metronidazole or tinidazole with a single-dose regimen recommended for most patients including pregnant patients. Those with HIV or failed initial treatment will require a longer course.^14^

Meningitis secondary to *N. meningitidis* is usually seen in young, previously healthy individuals who live in crowded environments.^5^ *N. meningitidis* is usually isolated in the nasopharynx of these individuals before becoming aerosolized and infecting close contacts.^6^ There have been case reports of *N. meningitidis* causing self-limited vaginitis, bacteremia, and arthritis after isolation from the genitourinary region. Our case demonstrates another case of symptomatic vaginitis from this organism; however, significant subsequent meningitis and septic shock ensued.

Despite a few case reports of *N. meningitides* isolation from the urogenital region of women previously, no current treatment recommendations exist.^2^ Although some of these reports showed spontaneous clearance, others describe empiric treatment with penicillin and amoxicillin yielding clearance of symptoms and subsequent negative cultures.^4^,^15^ However, resistance patterns have since changed; in the U.S. standard therapy against *N. meningitidis* generally consists of third-generation cephalosporins (e.g., cefotaxime, ceftriaxone). Resistance does not seem to have developed.^16^ Therefore, it seems reasonable to initiate therapy with these agents as well as obtain infectious disease consultation to help discern duration of treatment and ensure proper follow-up.

## CONCLUSION

Emergency physicians are often tasked with following up on culture results obtained previously in the ED or as an outpatient. This case of an uncommon isolate from a genitourinary culture highlights the possibility for subsequent invasive infection. While no true guidelines exist, we have outlined the available case reports of other patients presenting with varying degrees of symptomatology. We believe it is prudent for physicians to treat these findings aggressively and to stress the importance of follow-up for clearance of this organism in the otherwise systemically well patient.
